# Treat-to-Target and Regular Surveillance of Inflammatory Bowel Disease Are Associated with Low Incidence and Early-Stage Detection of Malignancies: A Retrospective Cohort Study

**DOI:** 10.3390/cancers15245754

**Published:** 2023-12-08

**Authors:** Tommaso Lorenzo Parigi, Mariangela Allocca, Federica Furfaro, Ferdinando D’Amico, Alessandra Zilli, Arianna Dal Buono, Roberto Gabbiadini, Stefanos Bonovas, Alessandro Armuzzi, Silvio Danese, Gionata Fiorino

**Affiliations:** 1 Department of Gastroenterology and Digestive Endoscopy, IRCCS Ospedale San Raffaele, 20132 Milan, Italyallocca.mariangela@hsr.it (M.A.); furfaro.federica@hsr.it (F.F.); damico.ferdinando@hsr.it (F.D.); zilli.alessandra@hsr.it (A.Z.);; 2 Division of Immunology, Transplantation and Infectious Disease, Università Vita-Salute San Raffaele, 20132 Milan, Italy; 3 Department of Biomedical Sciences, Humanitas University, Pieve Emanuele, 20072 Milan, Italy; 4 IBD Center, Department of Gastroenterology, Humanitas Research Hospital, Rozzano, 20089 Milan, Italy; arianna.dalbuono@humanitas.it (A.D.B.);; 5 IBD Unit, Department of Gastroenterology and Digestive Endoscopy, San Camillo-Forlanini Hospital, 00152 Rome, Italy

**Keywords:** inflammatory bowel disease, ulcerative colitis, Crohn’s disease, malignancy, cancer, colorectal cancer, surveillance, treat-to-target

## Abstract

**Simple Summary:**

Inflammatory bowel disease (IBD) increases the risk of cancer, particularly of the gastrointestinal tract. Modern management of IBD including a low threshold for acceptable inflammation (treat-to-target approach) and strict surveillance are believed to have reduced the incidence of malignancies associated with IBD. We conducted a retrospective study in two tertiary referral centers in Italy to evaluate incidence rates of all malignancies and colorectal cancer in patients with IBD and compare them with the general population. We observed incidence rates for all cancers and for colorectal cancer similar to that of the general population, and early-stage detection of malignancies through surveillance.

**Abstract:**

Inflammatory bowel diseases (IBD), including ulcerative colitis (UC) and Crohn’s disease (CD), increase the risk of malignancies, particularly colorectal cancer (CRC). We aimed to assess the incidence of malignancies in IBD patients managed using a treat-to-target approach and recommended surveillance. We retrospectively searched the electronic databases of two tertiary IBD centers in Milan from 2010 to 2019 for new diagnoses of malignancy in patients with pre-existing IBD. A total of 5239 patients with a follow-up of 19,820 years were included. In total, 71 malignancies were diagnosed in 70 patients (38 CD, 32 UC) with a mean age of 52.9 years, of whom 64% were former or active smokers. The annual incidence of all malignancies was 358 per 100,000 patient years (95% CI 275–444), and the standardized incidence rate (SIR) was 0.93 (95% CI 0.73–1.16). Gastrointestinal cancers were the most frequent (*n* = 17, 23.9%), in particular, CRC (*n* = 9), with an incidence of 45 per 100,000 (95% CI 15–74) and an SIR of 1.18 (95% CI 0.54–2.09). CRC occurred mainly in UC patients (6/8), while small bowel cancer was seen in CD patients (5/9). Melanoma and breast cancer (*n* = 8 each) were the most common non-GI cancers. No significant difference in incidence was found between CD or UC. Death occurred in nine patients (11%) and was due to cancer in eight of these cases, two of which were IBD-related. Most malignancies included in the surveillance were diagnosed at early (I–II) stages (20 vs. 4, *p* < 0.05). In patients with IBD, treat-to-target and strict surveillance were associated with a low incidence of cancer, similar to that of the general population, and the detection of malignancies at an early stage.

## 1. Introduction

Crohn’s disease and ulcerative colitis, the two main types of inflammatory bowel disease (IBD), are chronic inflammatory conditions of the gastrointestinal tract that affect millions worldwide [1,2]. Patients with IBD are at increased risk of developing cancers of the gastrointestinal tract and particularly colorectal cancer (CRC), and this risk is proportional to the severity, extent, and duration of the intestinal condition [3,4,5]. Other cancers of the digestive tract with increased incidence in IBD are cholangiocarcinoma [6,7,8], especially in patients with UC, and primary sclerosing cholangitis (PSC) and cancer of the small bowel in patients with CD [9,10,11]. In addition to the disease-related risk of malignancies, some classes of drugs used in IBD have been linked to cancer. Most IBD medications function by suppressing or modulating the immune system. This is beneficial for managing the disease but, theoretically, it could also weaken the body’s ability to detect and fight cancer cells, potentially leading to malignancies. Among the drugs approved for IBD, the only ones with a consistent, albeit modest, association with malignancy are thiopurine and anti-TNF, with lympho- and myeloproliferative disorders and non-melanoma skin cancer [7,12]. By contrast, many other malignancies (i.e., cervical cancer [13,14]) and nearly all classes of treatments have been postulated as being linked, but the evidence for these links is conflicting or inconclusive [15]. Altogether, the main driver of oncologic risk in IBD is gut inflammation, followed to a lesser extent by systemic inflammation [16,17] and exposure to some drugs. These factors add up and result in an overall increased risk of cancer.

To mitigate the risk of cancer, various preventive measures have been put in place including screening for precancerous lesions, tailoring treatment to minimize inflammation, vaccination, and the promotion of a healthy lifestyle [18]. Patients with IBD followed up at the IBD centers at the Humanitas Research Hospital and at San Raffaele Hospital in Milan are managed and monitored strictly following the European Crohn’s and Colitis Organisation (ECCO) guidelines [7,19,20] and the STRIDE recommendations [21]. The purpose of this strict monitoring and treatment is to minimize long-term complications of IBD including the incidence of cancer and mortality. In this retrospective study, we aimed to investigate the incidence of malignancy and mortality in the two centers and compare it with that expected from other IBD cohorts published in the literature and in the general population.

## 2. Materials and Methods

### 2.1. Follow-Up

All patients with UC extending proximal to the rectum and CD involving more than ⅓ of the colon undergo surveillance endoscopies every 1 to 3 years depending on risk factors, starting at 8 years from the onset of symptoms, or at diagnosis in cases of concomitant primary sclerosing cholangitis (PSC). Colonoscopy is performed by IBD-focused endoscopists using high-definition scopes and dye-based chromoendoscopy. All patients on azathioprine or advanced therapies undergo yearly dermatological visits, and females aged 25 to 64 undergo a 3-yearly PAP test and HPV vaccination. Every patient is advised to be vaccinated on their first visit to our IBD clinics, according to the ECCO recommendations and local guidelines. Patients with Crohn’s disease and also, since 2018, patients with ulcerative colitis are monitored yearly using magnetic resonance imaging and/or bowel ultrasound. Furthermore, in accordance with hospital guidelines, all patients with ileo-anal pouch anastomosis for IBD undergo a yearly pouchoscopy irrespective of risk factors. The two centers also adopt a uniform strategy of tight monitoring and treat-to-target, based on the STRIDE recommendations, with scheduled assessments of disease activity and escalation of therapies aiming at deep remission and complete mucosal healing.

### 2.2. Methods

We retrospectively searched the electronic database of the IBD Centre of the Humanitas Research Hospital and San Raffaele Hospital, which are both in Milan, Italy, from January 2010 to October 2019. We included in the analysis only patients with a confirmed diagnosis of IBD and at least two visits to the IBD center, whereas we excluded patients with only one visit or without an established diagnosis of IBD. For each patient, the follow-up was considered the interval from the first to the last visit to the center.

We recorded all new diagnoses of solid and hematological malignancies that occurred during follow-up and at least 6 months after the diagnosis of IBD. Only primary malignancies were included, whereas recurrences of previously diagnosed cancers were excluded. Data on cancer location, type, and staging at the time of diagnosis were recorded from the clinical notes, as well as patients’ demographics, smoking habits, and disease characteristics.

The annual cancer incidence and cancer-related mortality were calculated by dividing the number of incident cases or deaths by the total years of follow-up. The standardized incidence rate was obtained by dividing observed incidence by expected incidence. Confidence intervals were calculated based on Poisson distribution. Descriptive statistics were used to present characteristics, and parametric and non-parametric tests were applied to compare variables between groups. The analysis was performed with Excel (Office Mac version 16.78, Microsoft, Redmond, WA, USA).

## 3. Results

### 3.1. Study Population

The databases comprised 6548 patients. After filtering for confirmed diagnosis of IBD and at least two visits to the center, 5239 patients were included, with a total follow-up of 19,820 years (mean 3.7 years, range 0.5–9.8 years).

The average age was 40.4 (SD 15.0) with a mean disease duration of 8.7 years; 48% of patients were females; 58.9% had a diagnosis of UC and 40.9% a diagnosis of CD. Demographics and disease characteristics are presented in Table 1.

### 3.2. Malignancies

In the study population, 84 malignancies in 81 patients were identified, of which 71 were included in the analysis; 13 were excluded because they predated the diagnosis of IBD or presented in the first 6 months after IBD diagnosis.

The 71 included cancers occurred in 70 patients (38 CD and 32 UC), 31 (44%) of whom were females. The average age at tumor diagnosis was 52.9 years (range 19–78), significantly higher than that of the general population (*p* < 0.05); 64% of cancer patients were former or active smokers, and 31% had a family history of cancer.

With regard to treatment exposure, 62% of patients were being treated or had been treated with aminosalicylates, 40% with azathioprine or methotrexate, and 43% with a biologic (anti-TNF, vedolizumab, or ustekinumab) or with advanced drugs in clinical trials. Treatments are not mutually exclusive; hence, the sum of the percentages is greater than 100. The demographics and characteristics of patients who developed cancer are summarized in Table 2.

The annual incidence rate for all types of malignancies was 358 per 100,000 patient years (71/19,820; 95% CI 275–444). Based on Italian National Cancer Registry data, the resulting standardized incidence rate for all cancers was 0.93 (95% CI 0.73–1.16).

The annual incidence of CRC in IBD patients was 45 per 100,000 patient years (9/19,820; 95% CI 15–74), and 57 per 100,000 patient years (6/10,460; 95% CI 21–124) in patients with UC. The standardized incidence rates were 1.18 (95% CI 0.54–2.09) and 1.49 (95% CI 1.00–1.98), respectively (Table 3).

### 3.3. Types of Malignancies

Malignancies of the gastrointestinal (GI) tract were the most common (*n* = 17, 23.9%), distributed roughly evenly between UC (*n* = 8) and CD (*n* = 9). As expected from the literature, the majority of GI malignancies in UC patients (6/8 or 75%) were diagnosed in the colon or rectum in patients with a long disease duration (mean 22.5 years). By contrast, five out of nine GI cancers detected in CD (56%) were located in the small bowel, a tract seldom affected by cancer in the general population.

Melanoma and breast cancer (*n* = 8 each) were the most common non-GI malignancies diagnosed, followed by prostate (*n* = 7) and bladder (*n* = 6). Other incident tumors were thyroid (*n* = 5), lung (*n* = 4), testicle (*n* = 3), ovary (*n* = 2), kidney (*n* = 2), head–nose–throat (*n* = 2), pancreas (*n* = 1), brain (*n* = 1), and non-melanoma skin cancer (*n* = 1) (Table 4).

The incidence of all cancers was not significantly different between CD and UC. Comparisons for single types of cancer were not sufficiently powered; however, hematological malignancies and non-Hodgkin lymphomas and leukemia (3 and 1, respectively) only occurred in CD patients.

Cancers included in a screening protocol (small bowel, colon, rectum, anus, hematological, and skin cancers) were detected significantly more at early stages (I–II) than at advanced (III–IV) stages (20 vs. 4, *p* < 0.05), although no differences in mortality rates were reported between the two groups (2 vs. 2) (Table 5).

### 3.4. Mortality

Nine patients died during follow-up; eight died due to advanced cancer and one due to ischemic heart disease. The eight cancer-related deaths were due to lung (3), ovarian (2), pancreatic (1), colorectal (1), and anal cancer (1). The latter two, colorectal and anal cancers, were considered to be related to the concomitant IBD. Importantly, both IBD-related cancers were diagnosed at an advanced stage. The revaluation of the individual cases confirmed that both patients had long gaps in their endoscopy surveillance histories.

## 4. Discussion

Cancer, and particularly CRC, is a rare but fearful complication of IBD. The increased risk of malignancies in IBD patients is mainly attributed to chronic inflammation and, to a lesser degree, to the immunosuppression induced by the treatments for IDB [22]. To mitigate the risk of CRC, endoscopic surveillance is recommended in patients with at least one third of the colon involved, starting 8 years after diagnosis with intervals based on additional risk factors, such as extension of the disease, previous dysplasia, strictures, the presence of numerous pseudopolyps, a family history of CRC, or concomitant PSC [23,24]. Screening measures for other malignancies associated with IBD are less codified. In the two centers participating in this study, on top of routine blood tests, yearly dermatological visits were required for patients on immunomodulator or advanced treatments, and yearly small bowel imaging either with bowel ultrasound or magnetic resonance was offered to all patients with CD and, starting from 2018, to those with UC too.

In CRC, the severity of gut inflammation is directly correlated to the risk of cancer, and this is true even for mild disease activity evident only at histology [25]. Over recent decades, more effective medications have become available, the targets of treatment have gradually increased, stricter endoscopic surveillance strategies have been implemented, and the image resolution of endoscopes has improved. All these factors are believed to have led to a reduction in CRC risk in patients with IBD. This hypothesis is supported by numerous studies including large nationwide Scandinavian registries [26,27] that show a steady decline in the incidence of CRC and mortality in patients with IBD over time [28,29,30,31]. We may extrapolate from this that the evidence suggests that the better the care, the lower the cancer incidence and the earlier the detection.

Our retrospective study investigated the incidence of all malignancies in IBD patients under a particularly strict policy for both treatment and surveillance. The standardized incidence rate for all cancers was 0.93 (95% CI 0.73–1.16), similar to the incidence expected in the general population, suggesting that IBD was not associated with greater risk [32].

With regard to colorectal cancer, the incidence observed in our cohort (45 cases every 100,000 patient years) was considerably lower than that expected from previous studies [33,34]. As a reference, the Swedish and Danish IBD national registries recorded an incidence of CRC of 550 and 470 per 100,000 patient years in UC and CD patients, respectively [26,27]. However, these figures are calculated over a time period of nearly 50 years (1969–2017), through which IBD management changed dramatically, and they do not capture the specificities of IBD management in specialized centers. Moreover, these same nationwide studies observed a roughly 50% drop in the hazard ratios of CRC from the first to the latest decade considered in the analysis, also suggesting that incidence in recent years was considerably lower. On the other hand, in the general Italian population, the incidence rate of CRC between 1990 and 2017 was 38.1 per 100,000 patient years, only modestly lower than that observed in our IBD (45/100,000; 95% CI 15–74) and UC cohorts (57/100,000; 95% CI 21–124). These comparisons should be taken with caution since the distribution of risk factors may vary. Nevertheless, it is reassuring that in a cohort of IBD patients managed according to the latest recommendations, the incidence of CRC is only marginally higher than that in the general population.

The difference in CRC risk between historical national IBD registries and our experience is one of the main findings of this study. Our work was conducted on data from recent years, 2010–2019, when advanced medications and non-invasive monitoring were widely available. Both the centers at the time of this study were equipped with high-definition scopes, and IBD patients were managed by gastroenterologists fully dedicated to IBD, ensuring uniform, state-of-the-art, evidence-based practice. We believe that, altogether, these improvements are responsible for the reduction in excess cancer. Consistent with the broader trend of diminished CRC risk, published data on cancer stage at the time of diagnosis, although indirect, point to a reduction in advanced-stage cancer diagnosis in more recent cohorts [35]. In line with this, our study found that only 2 out of 28 malignancies included in some form of surveillance were diagnosed at advanced (III–IV) stage. Two more, which resulted in death, were diagnosed at late stage due to missed surveillance, a pattern confirmed in larger studies [36]. Once more, our results support the hypothesis that modern proactive management and surveillance of IBD significantly reduces cancer risk and shifts detection in favor of early-stage cancers.

The anatomical distribution of gastrointestinal cancers reflects the pattern of bowel involvement of UC and CD. Small bowel cancer, a rare malignancy in the general population, was observed only in CD patients, confirming various previous studies reporting a strong epidemiological link between the two conditions [37], while CRC was more common in patients with UC, especially in cases of long-standing disease.

The particularly low incidence of CRC supports the notion that targeting mucosal healing and escalating treatment to achieve it removes the main risk factor for cancer development. As treat-to-target strategies are increasingly implemented in clinical practice we expect our results to be confirmed in other cohorts supporting a broader rescaling of CRC risk in IBD.

The main strength of our study is the relatively large population homogeneously surveilled according to ECCO recommendations and treated with advanced therapies in two tertiary referral centers. Our results allow us to draw conclusions on incidence and mortality rates in specialized centers, a setting different from national registries. In fact, as care for IBD increases in complexity and is progressively centralized in expert centers, there is a need for updated epidemiological data that are more reflective of tertiary care. The relatively short time frame (2010–2019) limits the changes in medical and surveillance practices, reducing the variability attributable to newer treatments or evolving endoscopy techniques. At the same time, the sample size and the low overall number of malignancies limit the investigation of associations with specific patient characteristics and medication. In particular, the possible link with therapy is as interesting as it is challenging to study. In fact, very large datasets would be needed to isolate the hypothetical effect of a single drug from medical histories spanning years. Despite the difficulties, the available evidence consistently points to no increase in the risk of cancer in cases where advanced agents are used [38,39,40,41], although the data on newer medications such as small molecules are limited [42,43]. Our analysis spans from 2010 to 2019, a time when, in Italy, the only advanced treatments approved for IBD were infliximab, adalimumab, golimumab, vedolizumab, and ustekinumab. The safety profile of these treatments is now well established and their risk of malignancy reassuring. Interestingly, a recent Japanese nationwide study including over a thousand IBD-associated cancers found that use of biologics was associated with earlier-stage, rather than advanced-stage, cancer in UC patients [35]. Although the limited sample size of our cohort impeded a formal analysis, it is worth noting that three out of four patients who developed a hematologic malignancy were being, or had been, treated with azathioprine, which is known to increase the risk of lymphoproliferative disorders [44,45,46]. Another limitation is that this study did not evaluate the quality parameters of endoscopy or the type of surveillance practice. Several variables including cecal intubation rates, bowel preparation, and withdrawal time are known to affect neoplasia detection and post-colonoscopy CRC [47,48]. In addition, the use of random biopsies and dye-spray or digital chromoendoscopy, especially in high-risk patients, could improve diagnostic yield. Unfortunately, because our study was based on the electronic medical records of the clinic visits, these details were not available.

Finally, the inevitable limitations due to the retrospective nature of this study should be acknowledged, in particular the risk of selection bias and incomplete data. Given the importance of cancer diagnosis, it is unlikely that such information would have been omitted from the visit record. In the event that cancer disrupted gastroenterological follow-up and the patient missed or canceled his/her appointments, the diagnosis would still have been retrieved from non-gastroenterological records, provided these were from the institution. In other words, we cannot exclude the possibility that an IBD patient from our centers, hence, one included in the at-risk population, developed cancer and sought care elsewhere without ever returning to the hospital, an eventuality that would have led to an unreported diagnosis. However, we believe this risk is negligible, and it should not have impacted the overall result.

## 5. Conclusions

In two large IBD centers strictly applying recommended surveillance and treat-to-target strategies, cancer incidence was lower than expected from the literature and similar to that among the general population. In addition, malignancies diagnosed through surveillance were detected at early stages. These results support the usefulness of treat-to-target and the recommendation of surveillance in reducing cancer risk and facilitating early detection.

## Figures and Tables

**Table 1:** Study population.

Patient Characteristics	Number
Total population	5239
Total person-years	19,820
Female	2515 (48%)
IBD type	
UC	3083 (58.9%)
CD	2142 (40.9%)
IBD-U	13 (0.2%)
Age mean (SD)	40.4 (15.0)

**Table 2:** Demographics and characteristics of IBD patients who developed cancer.

Characteristics of IBD Patients Who Developed Cancer	
	Number (%) (Range)
Age at cancer diagnosis	52.9 (19–78)
Sex	Male 39 (55.7)
	Female 31 (44.3)
IBD type	UC 32 (45.7)
	CD 38 (54.3)
Drug exposure	Aminosalicylates 62%
	Antimetabolite 40%
	Biologics 43%
Family * history of cancer	31%
Smoker (past or current)	64%

IBD: inflammatory bowel disease; UC: ulcerative colitis; CD: Crohn’s disease. * Family defined as first-degree relatives.

**Table 3:** Incidence and standardized incidence of cancer in the IBD cohort.

		Cancer Incidence	
	Malignancies	Incidence Rate per 100,000 Patient Years	Standardized Incidence Rate (SIR)
All cancers	71	358 (95% CI 275–444)	0.93 (95% CI 0.73–1.16)
CRC	9	45 (95% CI 15–74)	1.18 (95% CI 0.54–2.09)
CRC in UC	6	57 (95% CI 21–124)	1.49 (95% CI 1.00–1.98)

CRC: colorectal cancer; UC: ulcerative colitis.

**Table 4:** Types of malignancies.

	Total	UC	CD
Gastrointestinal	17 (24%)	8	9
Stomach		2	
Small bowel			5
Colon and rectum		6	3
Anus			1
Melanoma	8 (11.2%)	4	4
Breast	8 (11.2%)	4	4
Prostate	7 (9.9%)	5	2
Bladder	6 (8.4%)	3	3
Thyroid	5 (7.0%)	2	3
Hematologic	4 (5.6%)		4
Lung	4 (5.6%)	2	2
Testicle	3 (4.2%)	2	1
Ovary	2 (2.8%)	1	1
Kidney	2 (2.8%)	1	1
Head–nose–throat	2 (2.8%)	1	1
Pancreas	1 (1.4%)		1
Non-melanoma skin cancer	1 (1.4%)	1	

**Table 5:** Stage of cancer diagnosis for malignancies included in surveillance or screening.

	Early (I–II) Stage	Advanced Stage (III–IV)
Small bowel	4	1
Colon and rectum	7	2
Anus	0	1
Melanoma	4	0
Non-melanoma skin cancer	1	0
Hematologic	4	0

## Data Availability

The data supporting this study can be requested from the corresponding author.
